# Generation of reactive oxygen species by hydroxypyridone compound/iron complexes

**DOI:** 10.1080/13510002.2020.1787662

**Published:** 2020-07-02

**Authors:** Keiko Murakami, Masataka Yoshino

**Affiliations:** Department of Biochemistry, Aichi Medical University School of Medicine, Nagakute, Japan

**Keywords:** Hydroxypyridone, mimosine‌, deferiprone, iron, reactive oxygen species, DNA damage, hydrogen peroxide‌, superoxide

## Abstract

**Objectives:** Prooxidant properties of iron-binding hydroxypyridone compounds including deferiprone and mimosine were analyzed.

**Methods:** Hydroxypyridone/iron-dependent production of reactive oxygen species was evidenced by the inactivation of aconitase, the most sensitive enzyme to oxidative stress in permeabilized yeast cells.

**Results and Discussion:** Deferiprone and mimosine produced reactive oxygen species in the presence of ferrous sulfate. The inactivation required sodium azide the inhibitor of catalase, and addition of TEMPOL, a scavenger of superoxide radical, protected aconitase from the inactivation, suggesting that the superoxide radical produced from the hydroxypyridone/iron complex is responsible for the inactivation of aconitase. A principal role of superoxide radical was further supported by the finding that the hydroxypyridone/iron complex can inactivate aconitase in the presence of cyanide the inhibitor of superoxide dismutase. Deferiprone and mimosine stimulated the Fe^2+^ oxidation, resulting in the one-electron reduction of oxygen to form superoxide anion, which can inactivate aconitase by oxidizing the prosthetic iron-sulfur cluster. Mimosine further stimulated the ascorbate/iron-dependent formation of 8-hydroxy-2′-deoxyguanosine in DNA.

**Conclusion:** Biological toxicity of mimosine and deferiprone reported previously can be accounted for by the prooxidant properties of hydroxypyridone compounds: coordination complex with iron generates reactive oxygen species resulting in the disturbance of mitochondrial energy metabolism and DNA damage.

## Highlights

- Hydroxypyridones such as deferiprone and mimosine/iron complex produced reactive oxygen species.
- Generation of reactive oxygen species was confirmed by the inactivation of aconitase.
- Deferiprone and mimosine produced the 8-hydroxy-2′-deoxyguanosine in DNA.
- Oxidation of ferrous ion was stimulated by deferiprone and mimosine.
- One-electron reduction of dioxygen produced superoxide radical.

## Introduction

**Figure 1.**
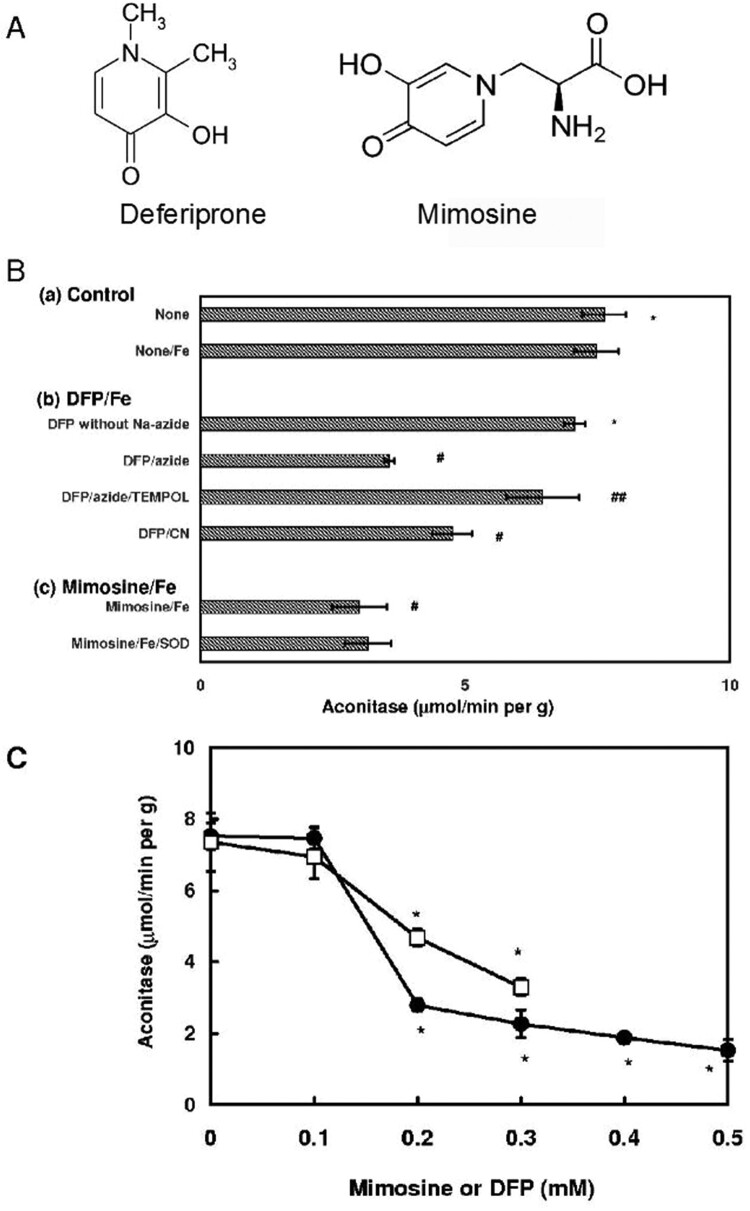
Effects of hydroxypyridone compounds on the activity of aconitase in baker’s yeast. (A) Structure of deferiprone and mimosine. (B) Effect of deferiprone and mimosine on the activity of aconitase. Permeabilized yeast cells were prepared according to the method reported previously [18], and the conditions of the aconitase inactivation were described in Materials and Methods. Incubation mixture for the aconitase inactivation contained 1mM TEMPOL or 50 μg/mL of superoxide dismutase (SOD). After incubation at 37°C for 10 min, cells were collected, and the aconitase activity was determined by the coupling with NADP-isocitrate dehydrogenase. Reaction mixture contained 5 mM citrate, 0.25 mM NADP, 4 mM MgCl_2_, 10 mU/mL of NADP-isocitrate dehydrogenase and 10 mg/mL of yeast. The increase in the absorbance at 340 nm was recorded. (a) Control group. (b) Deferiprone/Fe/sodium azide or KCN in the absence or presence of TEMPOL. (c) Mimosine/Fe/sodium azide in the absence or presence of superoxide dismutase. Results were represented as the mean ± SD, and analyzed using Dunnett’s test. * N.S. (statistically not significant difference) between the control groups (None and none/Fe). #, *p *< 0.01, a statistically significant difference between DFP/Fe/azide, DFP/Fe/cyanide or mimosine/Fe/azide and the control group. ##, *p *< 0.01, the DFP/Fe/azide/TEMPOL compared with DFP/Fe/azide group. C. Effect of varying concentrations of deferiprone and mimosine on the inactivation of aconitase in permeabilized yeast cells. Experimental conditions were similar to those described in section B. •, mimosine; □, deferiprone. Results were represented as the mean ± SD, and analyzed using Dunnett’s test. * *P *< 0.01 compared with control (without deferiprone).

Reactive oxygen species are closely related to various pathological conditions such as ischemia/reperfusion [1], and can be often generated depending on the redox state of transition metals [2]. Various compounds that bind metals can act as scavengers of reactive oxygen species: deferiprone (3-hydroxy-1,2-dimethyl-4[1H]-pyridone), one of the hydroxypyridone compounds (Figure 1(A)) can scavenge hydroxyl radical and superoxide anion [3,4] by binding iron potently [5], and has been applied for clinical use in diseases of systemic iron overload [6,7]. On the contrary, metal-binding properties of deferiprone causes an inactivation of mitochondrial aconitase by depleting iron of the enzyme active sites [8], and further results in the inhibition of the proliferation of skin fibroblasts and some tumor cells [9,10]. Mimosine ([2S]-2-amino-3-[3-hydroxy-4-oxopyridin-1-yl]propanoic acid) (Figure 1(A)) with a hydroxypyridone structure similar to deferiprone affects cell cycle progression [11]: feeding of large amount of mimosine leads to growth inhibition [12,13], and induction of apoptosis in cancer cells [14,15]. Some metal-binding compounds show cytotoxicity by the production of reactive oxygen species [16,17] in addition to the depletion of essential iron. Here, we examined the participation of the reactive oxygen species in the toxicity of hydroxypyridone compounds. Hydroxypyridone/iron complex produced reactive oxygen species, and the production was evidenced by the inactivation of aconitase, the most sensitive enzyme to oxidative stress, and the formation of DNA base adduct may account for the cytotoxicity of these coordination complexes.

## Materials and methods

### Materials

Deferiprone, mimosine, NADP, TEMPOL (4-hydroxy-2,2,6,6-tetramethylpiperidine-1-oxyl), bathophenanthroline disulfonate, calf thymus DNA, bovine erythrocyte superoxide dismutase, and ascorbic acid were products of Sigma-Aldrich-Japan (Tokyo, Japan). Yeast NADP-isocitrate dehydrogenase was purchased from Oriental Yeast Co. (Tokyo, Japan). Baker’s yeast was obtained locally.

### Incubation conditions and determination of the aconitase activity

Baker’s yeast was permeabilized with toluene [18], and used for analyzing the effect of deferiprone and mimosine on the aconitase activity. The cells (10 mg/mL) were incubated with 0.2 mM deferiprone or mimosine in the presence of 50 μM FeSO_4_ and 1 mM NaN_3_ or KCN in 40 mM Tris-HCl buffer (pH 7.1). TEMPOL (1 mM) or superoxide dismutase (SOD, 50 μg/mL) was included in the reaction mixture in order to confirm the role of superoxide racial for the inactivation of aconitase. After incubation at 37°C for 10 min, cells were collected by centrifugation at 800 × g for 5 min and suspended in 50 mM Tris-HCl (pH 7.1) containing 0.5 M sorbitol at the concentration of 200 mg/ml. Aconitase activity was determined spectrophotometrically by the increase in the absorbance of NADPH at 340 nm with the coupling method using NADP-isocitrate dehydrogenase [16,17]. Reaction mixture of 1 mL contained 5 mM citrate, 4 mM MgCl_2_, 0.25 mM NADP, 0.05 unit of purified NADP-isocitrate dehydrogenase from yeast and 50 mM Tris-HCl (pH 7.1). Reaction was started by the addition of 10 mg/mL permeabilized yeast cells. The increase in the absorbance at 340 nm was recorded.

### Autooxidation of ferrous ion

Interaction of deferiprone and mimosine with iron was evaluated by the determination of the enhanced rate of autooxidation of Fe^2+^ ion by these compounds as described previously [19]. The samples of 2 mL contained 10 mM Tris-HCl (pH 7.1), 0.1 mM FeSO_4_ and deferiprone or mimosine. The reaction was started by the addition of FeSO_4_. Aliquots of 0.2 mL were mixed with 0.1 mL of 1 mM bathophenanthroline disulfonate at appropriate intervals, and the absorbance at 540 nm was measured.

### Determination of 8-hydroxy-2′-deoxyguanosine (8-OHdG)

Iron-dependent formation of 8-hydroxy-2′-deoxyguanosine was carried out by HPLC-ECD method as described previously [20]. Calf thymus DNA was treated with 0.1 mM ascorbate, 0.1 mM FeCl_3_ and various concentrations of mimosine for 1 h, and 8-OHdG and deoxyguanosine (dG) were determined. Data are expressed as mean ± SD with three independent determinations.

### Statistical analysis

JMP5.1J (SAS Institute Inc) was used for statistical analysis of the data. Results were analyzed using Dunnett’s test.

## Results

A prosthetic iron-sulfur cluster [4Fe-4S]^2+^ at the active site of aconitase is readily oxidized by reactive oxygen species such as superoxide radical, hydrogen peroxide, and hydroxyl radical to form inactive [3Fe-4S]^1+^ cluster, and further degradation products, and thus, the determination of the aconitase activity is a good indicator of oxidative stress [21,22]. We examined the generation of reactive oxygen species by hydroxypyridone compounds including mimosine and deferiprone. As shown in Figure 1(B), deferiprone with ferrous ion inactivated aconitase: the inactivation of the enzyme absolutely required sodium azide the inhibitor of catalase, whereas ferrous ion or deferiprone alone did not affect the aconitase activity. Addition of TEMPOL, a superoxide radical scavenger [23], protected the enzyme from the deferiprone/iron/azide-dependent inactivation, suggesting that the superoxide radical is responsible for the oxidative inactivation of aconitase. Aconitase was further inactivated by deferiprone/ferrous ion complex in the presence of cyanide the inhibitor of superoxide dismutase (Figure 1(B,b)): these findings also indicate that superoxide radical can play a principal role in the deferiprone/iron-dependent inactivation.

Mimosine/ferrous ion complex also inactivated aconitase in the presence of sodium azide, but the addition of superoxide dismutase could not affect the inactivation of aconitase (Figure 1(B,c)). Thus, hydrogen peroxide produced from superoxide radical by superoxide dismutase was also suggested to show an inactivating effect.

We further examined the dependence of the aconitase inactivation on the concentrations of deferiprone and mimosine. The concentrations of deferiprone and mimosine/iron complexes required for 50% inactivation of aconitase, were about 0.15 and 0.2 mM, respectively (Figure 1(C))

**Figure 2.**
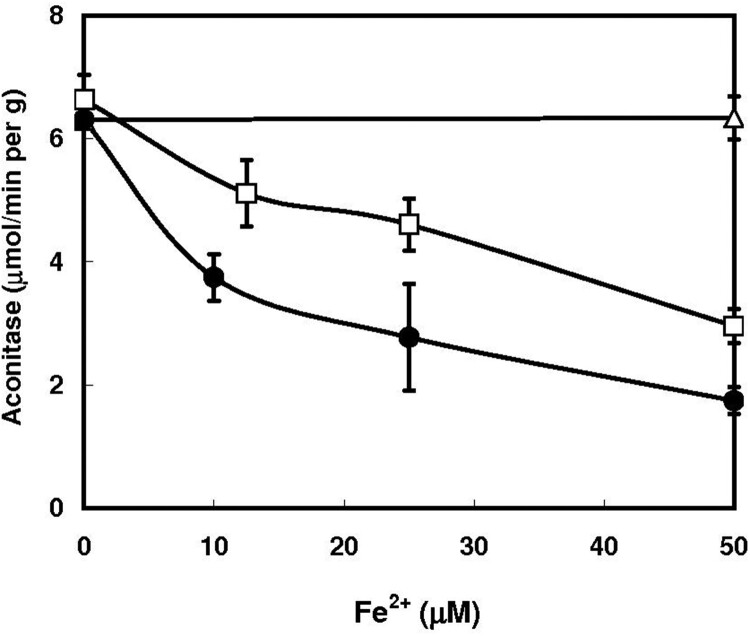
Effect of varying concentrations of ferrous sulfate on the inactivation of aconitase in permeabilized yeast cells. Experimental conditions were similar to those described in Figure 1, except that deferiprone concentration was kept at 0.5 mM, and FeSO_4_ concentrations were varied. ○, None; ●, 0.4 mM mimosine; □, 0.5 mM deferiprone.

Dependence of the hydroxypyridone-mediated inactivation of aconitase on the iron concentrations was examined. The concentrations of iron required for 50% inactivation of the enzyme were 40–50 μM (Figure 2).

**Figure 3.**
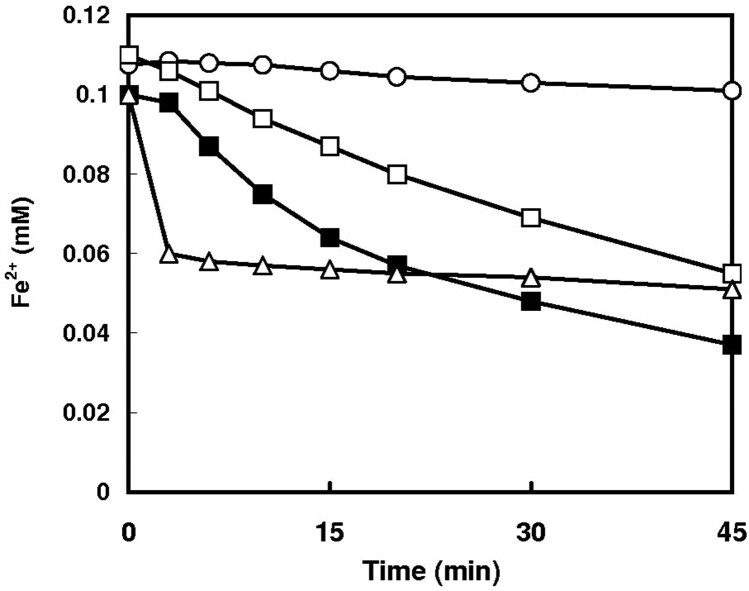
Effect of deferiprone and mimosine on the oxidation of ferrous ion. Iron oxidation of followed by determining the Fe^2+^ ion concentrations with bathophenanthroline disulfonate as described previously [19]. ○, none; □, 0.1 mM deferiprone; ▪, 0.2 mM deferiprone; Δ, 0.2 mM mimosine.

Oxidation of ferrous ion is closely related to the prooxidant action. We now examined the effect of hydroxypyridone compounds on the autooxidation of Fe^2+^ ion. Deferiprone stimulated the autooxidation of ferrous ion markedly, and mimosine also enhanced the Fe^2+^ autooxidation (Figure 3).

**Figure 4.**
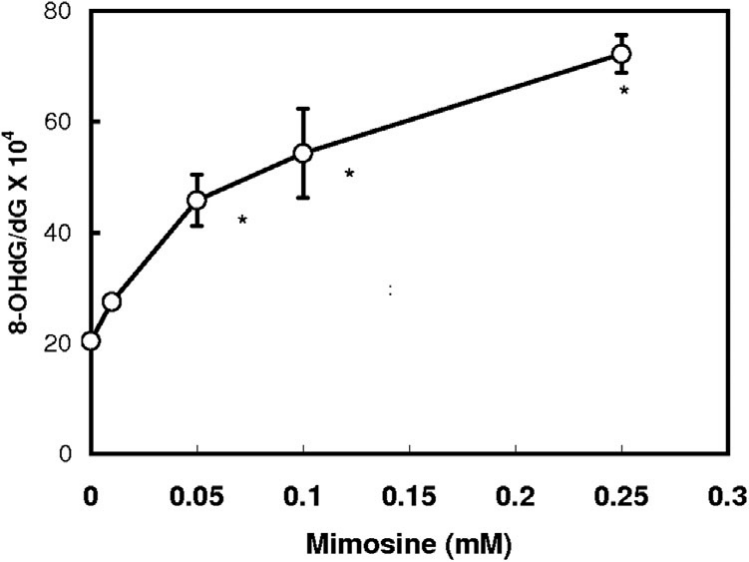
Effect of mimosine on the iron-dependent formation of 8-hydroxy-2′-deoxyguanosine in DNA. Iron-dependent formation of 8-hydroxy-2′-deoxyguanosine was carried out by HPLC-ECD method as described previously [20]. Calf thymus DNA was treated with 0.1 mM ascorbic acid and 0.1 mM FeCl_3_ in the presence of the indicated concentrations of mimosine for 1 h and 8-OHdG was determined. Data are expressed as mean ± SD with three different determinations. Asterisks indicate a significant difference in the 8-OHdG/dG ratio between the control and the mimosine/iron-treated group (*p *< 0.01).

Effect of mimosine on the oxidative damage of DNA was analyzed. When calf thymus DNA was treated with ascorbic acid in the presence of iron, 8-OHdG was effectively formed [20]. Addition of mimosine further increased the formation of 8-OHdG, and its concentration rose to a value 3.5 times as much by addition of 0.25 mM mimosine (Figure 4). However, a further increase in mimosine rather inhibited the formation of 8-OHdG (data not shown).

## Discussion

Hydroxypyridone compounds such as deferiprone and mimosine can act as an antioxidant by its potent iron-binding activity: for example, deferiprone binds to iron in a 3:1 (ligand: iron) molar ratio [5]. This compound can scavenge hydroxyl radical and superoxide anion [3,4], and thus, is used to treat iron overload in thalassemia [6,7]. On the contrary, deferiprone shows cytotoxicity such as an inactivation of aconitase by depletion of iron of the active site of the enzyme under certain conditions [8,9], and further causes an apoptotic cell death including DNA fragmentation and activation of caspase [24]. Mimosine, a tyrosine analog with deferiprone structure is a constituent of the tropical legumes *Leucaena* and *Mimosa*, which has been used as livestock feeds owing to high contents of protein, carotenoids, vitamin K and minerals, but feeding of large quantities of the plant to livestock leads to toxicity such as alopecia and growth inhibition [12]. These toxic effects and anti-neoplastic, anti-mitotic and apoptotic activities of mimosine [14,15] has been considered to be related to the potent metal-binding activity [11] causing the inhibition of iron-dependent enzymes belonging to DNA synthesis and TCA cycle [14,25–27]

The present study revealed that the cytotoxicity of hydroxypyridone resulted from the production of reactive oxygen species in the presence of iron. Production of reactive oxygen species, in particular superoxide radical, was verified by the inactivation of aconitase, one of the most sensitive enzymes to reactive oxygen species. Hydroxypyridone/iron-mediated inactivation of aconitase required sodium azide the inhibitor of catalase, suggesting that hydrogen peroxide or its precursor the superoxide radical is responsible for the inactivation. Of the reactive oxygen species produced, superoxide radical is presumed to be a principal role in the inactivation of aconitase because of its high rate constant for the reaction with the enzyme (10^7^ M^−1^ s^−1^)[21,22,28]. A principal role of superoxide radical was further proved by the protective effect of TEMPOL, a scavenger of superoxide radical [23], on the oxidative inactivation of aconitase. Inactivation of aconitase in the presence of cyanide the inhibitor of superoxide dismutase also supports the substantial role of superoxide radical in the inactivation. However, the cyanide-dependent inactivating effect was relatively weak compared with the azide-dependent inactivation (see Figure 1(B,b)). This difference can be accounted for by the action of the cyanide-insensitive Mn-superoxide dismutase in yeast. Cu/Zn-superoxide dismutase that can be inhibited by cyanide represents 80% of the total superoxide dismutase activity, and the Mn-superoxide dismutase with cyanide-insensitive properties represents approximately 20% of the total activity [29]. Thus, active Mn-superoxide dismutase can scavenge superoxide radical generated by deferiprone/iron complex, resulting in the deterioration of the aconitase inactivation, although Cu/Zn-superoxide dismutase reaction is inhibited under the conditions where only cyanide was included without sodium azide. Hydrogen peroxide can inactivate aconitase irrespective of its low rate constant (10^3^ M^−1^ s^−1^) under the experimental conditions where the inactivation reaction was carried out for 10 min (see, Figure 1(B)). Mitochondrial aconitase is demonstrated to be inactivated by hydrogen peroxide [30].

Generation of superoxide radical by hydroxypyridone/iron complex was closely related to the enhanced autooxidation of ferrous ion. Hydroxypyridone stimulated the Fe^2+^ oxidation, resulting in the one-electron reduction of oxygen to form superoxide anion, which can inactivate aconitase by oxidizing the prosthetic iron-sulfur cluster [4Fe-4S]^2+^ at the active site, producing the inactive [3Fe-4S]^1+^ enzymes with the release of Fe^2+^. Superoxide radical is dismutated into hydrogen peroxide by superoxide dismutase [31], and further hydrogen peroxide is converted to more reactive hydroxyl radical by the reduced transition metal-dependent Fenton reaction [32]. Hydroxyl radical causes further degradation of iron-sulfur cluster. Under the present experimental conditions, deferiprone-dependent oxidation of ferrous ion to ferric ion was comparable to the inactivation of aconitase: Ferrous ion oxidation and the aconitase inactivation were 32% and 40%, respectively (Figures 3 and 1(C)). Mimosine-mediated iron oxidation was also correlated with the inactivation of aconitase.

Concentrations of deferiprone and mimosine required for the 50% inactivation of aconitase were 150–200 μM, which were comparable to the concentrations necessary for the inhibition of proliferation of cancer cells: Addition of 150 μM deferiprone inhibits the proliferation of human cultured cell with the inactivation of mitochondrial aconitase [8]. Furthermore, the half and 90% inhibitory concentrations of deferiprone required for some cell lines are ranged within 51–67 μM and 81–486 μM, respectively [9].

**Figure 5.**
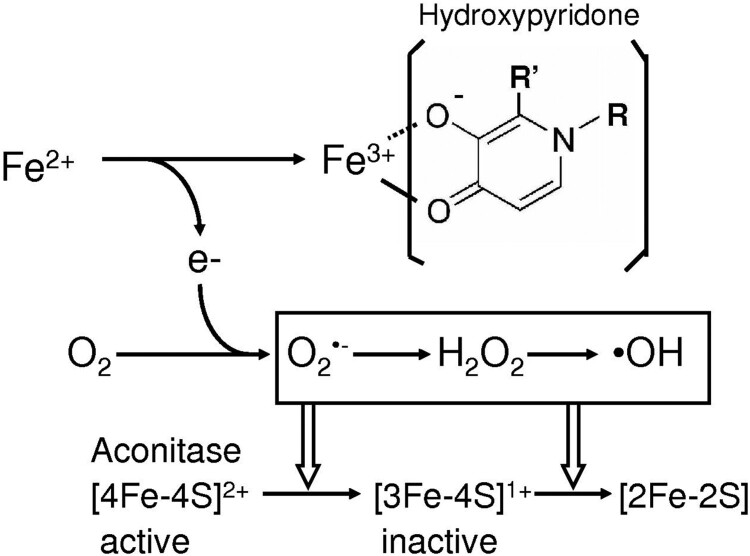
Hydroxypyridone/iron complex-dependent generation of reactive oxygen species.

Hydroxypyridone compounds such as deferiprone and mimosine with potent iron-binding activity act as an antioxidant, but administration of excess amount of these compounds shows toxicity including an inactivation of aconitase with mitochondrial dysfunction and decreased cell proliferation [8]. Growth retardation by mimosine [12] is closely relation to the DNA breaks in mammalian cells: the DNA damage is considered to be due to the mimosine-mediated production of hydroxyl radical [33–35]. In this work we demonstrated the hydroxypyridone/iron complex can generate reactive oxygen species and further stimulates the ascorbate/iron-mediated formation of 8-hydroxy-2′-deoxyguanosine. Hydroxyl radical-dependent formation of 8-hydroxy-2′-deoxyguanosine the base adduct may deteriorate cellular function. Cytotoxicity of mimosine and deferiprone can be concluded to be ascribed to the reactive oxygen species produced in the presence of iron (Figure 5).

## Conclusions

Present findings indicate that hydroxypyridone compounds such as mimosine and deferiprone as coordinating form with iron or copper can generate superoxide radical. Production of reactive oxygen species was analyzed by the inactivation of aconitase, the highly sensitive enzyme to oxidative stress, and further evidenced by the protective effect of TEMPOL against the oxidative inactivation of the enzyme. Further *in vivo* studies on the effects of mimosine and deferiprone may lead to understanding of biological toxicity of these compounds.
